# A Multi-Ingredient Containing Carbohydrate, Proteins L-Glutamine and L-Carnitine Attenuates Fatigue Perception with No Effect on Performance, Muscle Damage or Immunity in Soccer Players

**DOI:** 10.1371/journal.pone.0125188

**Published:** 2015-04-27

**Authors:** Fernando Naclerio, Eneko Larumbe-Zabala, Robert Cooper, Judith Allgrove, Conrad P. Earnest

**Affiliations:** 1 Center for Sport Sciences and Human Performance, University of Greenwich, Medway, United Kingdom; 2 Clinical Research Institute, Texas Tech University, Health Sciences Center, Lubbock, Texas, United States of America; 3 Faculty of Science, Engineering and Computing, Kingston University, London, United Kingdom; 4 Director of Research, Woodbolt International, and Texas A&M University, College Station, Texas, United States of America; University of Marburg, GERMANY

## Abstract

We investigated the effects of ingesting a multi-ingredient (53g carbohydrate, 14.5g whey protein, 5g glutamine, 1.5g L-carnitine-L-tartrate) supplement, carbohydrate only, or placebo on intermittent performance, perception of fatigue, immunity, and functional and metabolic markers of recovery. Sixteen amateur soccer players ingested their respective treatments before, during and after performing a 90-min intermittent repeated sprint test. Primary outcomes included time for a 90-min intermittent repeated sprint test (IRS) followed by eleven 15 m sprints. Measurements included creatine kinase, myoglobin, interleukine-6, Neutrophil; Lymphocytes and Monocyte before (pre), immediately after (post), 1h and 24h after exercise testing period. Overall, time for the IRS and 15 m sprints was not different between treatments. However, the perception of fatigue was attenuated (P<0.001) for the multi-ingredient (15.9±1.4) vs. placebo (17.8±1.4) but not for the carbohydrate (17.0±1.9) condition. Several changes in immune/inflammatory indices were noted as creatine kinase peaked at 24h while Interleukin-6 and myoglobin increased both immediately after and at 1h compared with baseline (P<0.05) for all three conditions. However, Myoglobin (P<0.05) was lower 1h post-exercise for the multi-ingredient (241.8±142.6 ng·ml^-1^) and CHO (265.4±187.8 ng·ml^-1^) vs. placebo (518.6±255.2 ng·ml^-1^). Carbohydrate also elicited lower neutrophil concentrations vs. multi-ingredient (3.9±1.5 10^9^/L vs. 4.9±1.8 10^9^/L, P = 0.016) and a reduced (P<0.05) monocytes count (0.36±0.09 10^9^/L) compared to both multi-ingredient (0.42±0.09 10^9^/L) and placebo (0.42±0.12 10^9^/L). In conclusion, multi-ingredient and carbohydrate supplements did not improve intermittent performance, inflammatory or immune function. However, both treatments did attenuate serum myoglobin, while only carbohydrate blunted post-exercise leukocytosis.

## Introduction

Intense and prolonged exercise leads to physiological stress and transient but clinically significant changes in immunity with elevations in stress hormones, pro- and anti-inflammatory cytokines, and reactive oxygen species [1]. This stress response induces the release of the noradrenaline, adrenaline, and cortisol/corticosterone, as well as a number of neurotransmitters, hormones, peptides, cytokines and other factors that potentially affect performance and the perception of fatigue perception [2]. Prolonged intermittent repeated-sprint-running exercise involving a high numbers of eccentric contractions as typically performed by soccer players has been associated with both transient structural muscular damage [3] and an alteration in the number and function of the innate immune system cells e.g. Neutrophils, Monocytes and Lymphocytes [2,4]. The exercise induced muscular damage and inflammatory response observed during, immediately post and throughout the recovery leads to the leakage of proteins and cytokines such as creatine kinase (CK) [5] Myoglobin (Mb) and interluekine-6 (IL-6) out of the cell and into the circulation [6]. Collectively, these responses may influence performance in sports involving intense, intermittent bouts of work.

Various nutritional interventions have been proposed to improve performance [7,8] attenuate the perception of fatigue [8,9]; muscle damage [10] immune dysfunction [11] or to enhance recovery [12] following training and intermittent sport competition. Alghannam (2011) [7] observed a positve effect on intermittent running performance after ingesting 0.3g·kg^-1^ and 0.7g·kg^-1^ of carbohydrate and protein in young male amateur soccer players. Similar effects were noted by Highton *et al*. (2013) [8]. While various studies have shown that combining carbohydrates with protein may improve performance [13], increase protein synthesis and enhance tissue repair [14], less is known about combining this nutrition schema with other ingredients. Accordingly, glutamine supplementation has been shown to attenuate fatigue [5] and aid immune recovery [15] while the ingestion of 1 to 2 g of L-carnitine L-tartrate has been associated with a decrease in exercise induced oxidative stress [16,17]. The examination of multi-ingredient formulae containing protein, carbohydrate and amino acid derivatives is important given recent reports attesting to an increased usage of such supplements among team sports athletes [18]. Posited benefits include an effort to support performance, reduce the perception of fatigue perception and maximize recovery between training and competitions [19,20]. Though scientific information is available, supplement users rely more on coaches, teammates, family and friends to obtain advice about nutrition products and protocols for consumption [21]. The final decision appears to be mainly based on the marketing claims, rather than on the available scientific information [22].

The primary aim of this investigation was to examine the effects of a commercially available multi-ingredient formulae composed of protein and carbohydrate, L-glutamine and L-carnitine L-tartrate versus a carbohydrate only (CHO) and placebo treatment on sprint performance, rate of perceived exertion, muscle damage, temporary immune dysfunction and recovery from intermittent protocol over 24h post exercise period in amateur soccer players. We hypothesized that the ingestion of a multi-ingredient would improve performance, attenuate perception of fatigue enhance recovery and reduce markers of muscle damage and immunosuppression compared to the ingestion of CHO alone or a placebo.

## Materials and Methods

### Participants

Sixteen males, amateur soccer players (age 24±3.7 years; height 181±1 cm; body mass 77.5±8.7kg) volunteered to participate in the study signed informed consent forms, and all study procedures previously approved by the Institutional Review Board at the University of Greenwich (UK). The study was conducted during the spring of 2013 at the Centre for Sport Sciences of Human Performance at the Medway Campus in Kent in accordance with the Declaration of Helsinki and approved by the University ethics committee. All participants were screened for any musculoskeletal injuries, metabolic conditions, or diseases; and use of medications, smoking, and nutritional supplements that would affect performance, rate of perceived exertion, muscle damage, immunology or recovery process (e.g., creatine, whey protein, and amino acids) within 6 weeks prior to the start of the study. A minimum sample size of 15 participants was calculated assuming a 0.05 significance level, a 0.80 power, and a correlation ≥0.70 among the 4 repeated measures within each condition, in order to detect medium effect sizes. We increased the required sample size up to 16, preventing from the possible attrition. Eventually, the initial sample completed the entire program.

### Study design

**Fig 1:**
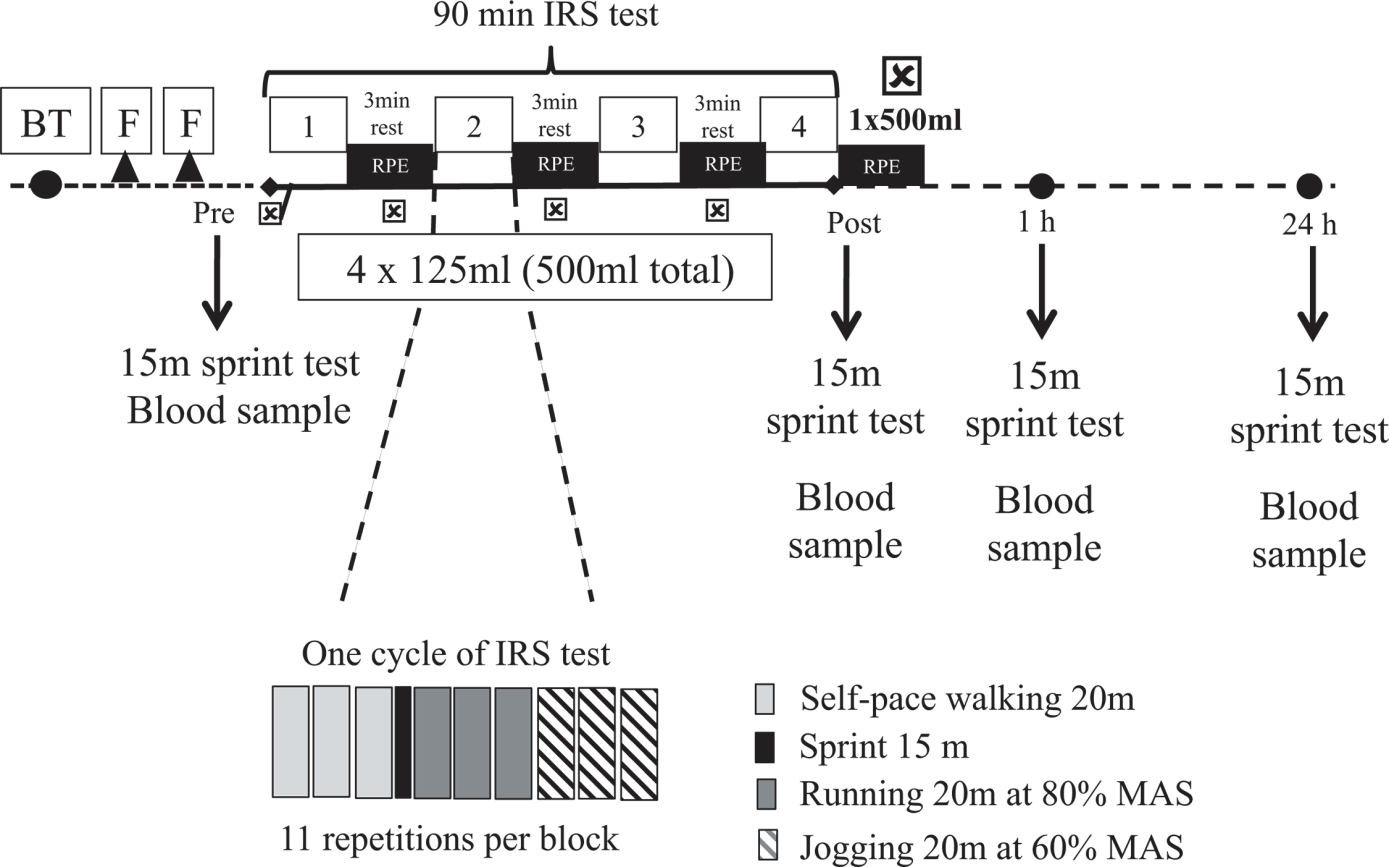
Schematic overview of the study design. BT beep test; F, familiarization Sessions, ☒ MTN, CHO, or PL ingestion; 90 min IRS test: intermittent repeated sprint test involving 4 blocks of 11 cycles of 3 repetitions of 20 m at 60%; one 15m sprint; 3 repetitions of 20 m at 80%, and at 60% maximal aerobic speed; RPE: determination of the rate of perceived exertion value from the 6–20 Borg scale points; 15m: sprint test; Blood: blood extraction from the cubital fossa.

We utilized a double blind, repeated measures, counter-balanced, crossover design, where participants ingested three supplement treatments: (1) multi-ingredient (MTN; protein (14.5 g), carbohydrate (53 g), L-glutamine (5 g), and L-carnitine L-tartrate (1.5 g), (2) carbohydrate (CHO; (69.5 g) and (3) placebo. Upon eligibility, participants were required to attend the laboratory on six different occasions. On the first visit participants were assessed for body mass, height, and maximal aerobic speed (MAS). The next two visits were used to familiarize participants with a 90-min intermittent repeated sprint test (IRS) [23] and the use of the rate of perceived exertion (RPE) (6–20) scale [24]. The remaining visits comprised the performance testing with each respective treatment with seven days allowed between each of the three testing conditions. Additionally, participants were asked to refrain from any unaccustomed or hard exercise during the 72-h before each of the three main testing sessions. Fig 1 shows the structure of study protocol.

### Procedures

Prior to each experimental condition, participants were required to provide a diet diary for three consecutive days consisting of two week days and one weekend day. Diets were then analysed for macronutrient composition using Dietplan6 software (Forestfield Software, UK). Participants were instructed to maintain their normal diet throughout the intervention.

### Pre-exercise standardized meal

In order to minimize large variability in energy intake and muscle glycogen concentrations, two hours before arriving to the laboratory, participants were required to consume a standardised meal sourced from porridge oats and semi-skimmed milk that provided 1 g·kg^−1^ CHO and 0.15 g·kg^−1^ protein.

### Determination of MAS

A standard multistage 20-m shuttle run test, starting at 8.5 km·h^−1^ and increasing by 0.5 km·h^−1^ every minute, was used to predict MAS [25]. The test was stopped when participants failed to arrive at the line before the audible beep on two consecutive occasions. Verbal encouragement was given and a minimum score of level 8 had to be achieved for a participant to be considered suitable for the study. Furthermore, MAS was estimated from the last completed stage and used to pace sections of the IRS.

### Intermittent Repeated Sprint Test

After obtaining a baseline blood sample and immediately prior to the IRS, participants performed a standardized five min warm-up involving different shuttle run speeds and dynamic stretching was completed. The IRS was a modified version of the Loughborough Intermittent Shuttle Test [26] of nearly 90 min in duration and divided into 4 blocks with 3 min of rest between each block. Each block consisted of 11 cycles of 3 repetitions of 20 m of self-paced walking followed by one 15-m sprint after which 3 repetitions of 20 m of running at 80% of MAS and 3 repetitions of 20 m of jogging at 60% of MAS were performed. Therefore, a total of 44 cycles were completed for each IRS, covering a total distance of 8580 m at varying velocities.

### Intermittent sprint performance

The sum of eleven 15-m sprint times obtained per each of the 4 blocks and from the total 44 sprints performed for entire IRS was considered as indicators of sprint performance.

### Rate of Perceived Exertion (RPE)

Borg scale (6–20) [24] was used to determine the RPE at the end of each block and after completing the IRS.

### Fifteen-meter sprint test

The 15-m sprint test was selected to specifically examine fatigue induced by the IRS [27]. Each participant performed three 15-m sprints; each sprint time was measured using an infrared timing gate system (Brower Timing Systems). After walking back to the start of the sprint track, participants were required to rest for 30 s between sprints to allow for recovery, and the best of the 3 sprints was used for the analysis. The coefficients of variation for this test, calculated from reliability trials conducted in previous pilot studies, are between 0.5% and 1%.

### Blood sampling and analysis

Fatigue assessment and biomarker measures were determined before the IRS (pre), immediately after IRS (post), 1h after IRS (1h), and 24h after IRS (24h).

Vacutainer venous blood collection tubes were used to collect 10 mL of heparinised blood and 10 mL of whole blood from the cubital fossa. An aliquot of the whole blood was used to perform leukocyte (Neutrophil; Lymphocytes and Monocyte) counts as general markers of immunity using an automated haematology analyser (ABX Pentra 60C+, Horiba Medical, Montpellier, France). Nonheparinised blood samples were inverted 5 times and allowed to stand for 1h prior to being centrifuged at 3500 r x min^–1^, at a temperature of 4°C, for 10 min, after which the serum was aliquoted into Eppendorf tubes and frozen at—70°C for later analysis. Heparinised samples were inverted 8 times in the vacutainer to ensure adequate mixing with the heparin. Thirty-two microlitres of heparinised blood was pipetted out onto a test strip and analysed, for CK, using a colorimetric assay procedure (Reflotron Boehringer Mannheim, Germany) (Horder et al. 1991). The remaining heparinised blood was centrifuged at 3500 r x min^–1^, at a temperature of 4°C, for 10 min, after which the plasma was aliquoted into Eppendorf tubes and frozen at—70°C for later analysis. IL-6 (R&D Systems; HS600B, Abingdon, UK) and Mb (Abcam; ab108652, Cambridge, UK) were each assayed in duplicate using an ELISA in accordance with the assay kit instructions provided by the manufacturer. As reported in the manufacturer’s data sheets, the intra-assay and inter-assay coefficients of variation for CK were 3.0% and 3.5%, respectively. For Mb assays, they were 3.5%–6.0% and 5.0%–10.0%, respectively. Coefficients of variation for IL-6 were 6% to 8% and 10% to 12%.

### Supplementation protocol

Immediately prior to the first, second, third, and fourth blocks of the IRS, participants ingested 500 mL of water mixed with MTN, CHO, or placebo that were divided into 4 equal doses. Each 500-mL dose of MTN contained 53 g of CHO (maltodextrin and dextrose), 14.5 g of whey protein, 1.2 g fat, 5 g of glutamine, 1.5 g of L-carnitine-L-tartrate, and provided 280 kcal, whilst a 500-mL dose of CHO contained 69.5 g of maltodextrin only and provided 265 kcal. The placebo was a low kcal beverage (20.97 kcal per serving) of the same volume, color, and flavor as MTN and CHO. A second full dose was provided 20 min after the IRST. A total of 2 full doses of MTN, CHO, or placebo were ingested in each condition. Therefore, the MTN supplement provided a total of 106 g CHO, 29 g protein, 2.4 g fat, 10 g glutamine, 3 g L-carnitine- L-tartrate, and 560 kcal, whilst the CHO supplement provided a total of 139 g CHO and 530 kcal.

### Statistical analysis

Mauchly’s Test of Sphericity was used for testing the normality distribution of the data. Two way repeated measures analysis of variance (ANOVA) (3 test conditions x 4 sprint blocks) was performed to analyse the total sprint time (sum of 44 sprints) performed at IRS. The differences between treatment conditions and time points were assessed using 2-way repeated measures ANOVA (3 test conditions x 4 time points). Bonferroni-adjusted post hoc analysis was performed for pairwise comparisons. To measure standardized effect size, eta squared (η^2^) was used. In absence of specific thresholds from the literature, reference values [small (η^2^ = 0.01), medium (η^2^ = 0.06), and large (η^2^ = 0.14)] from Cohen (1988) [28] were considered. Data are presented as Mean ± SD. Significance level was set at P ≤ 0.05 for all tests.

## Results

### Diets

Overall, participants consumed an average daily macronutrient intake of CHO (5.51±0.21 g·kg^−1^), protein (1.4±0.26, 1.15±0.16 g·kg^−1^, fat, (33.5±1.3 kcal·kg^−1^) and verbally confirmed that they maintained their habitual diet throughout the trial period.

### Repeated sprint performance

Table 1 shows the total times summarised at blocks 1, 2, 3, and 4, as well as for the total IRS for each of the three treatment conditions. A significant interaction effect was observed between treatment conditions and time per block (F_6,90_ = 2.19, P = 0.050, η^2^ = 0.01). No significant main effect was observed when comparing the sum of the total sprint time for the entire IRS per condition (F_2,30_ = 0.25, P = 0.782, η^2^≈0). Significant effects were observed when considering the time performed per block (1^st^, 2^nd^, 3^rd^, and 4^th^), F_3,45_ = 7.49, P<0.001, η^2^ = 0.01. Although significantly shorter times for block 1 compared to block 3 (P = 0.015) and 4 (P = 0.007) were identified for CHO condition, further post hoc analysis revealed no significant differences between treatment conditions.

**Table 1:** Total 15-m sprint times (s) per block and the entire intermittent repeated sprint test.

Condition	All 4 blocks (s)	Block 1 (s)	Block 2 (s)	Block 3 (s)	Bloc 4 (s)
MTN	107.9(9.3)	26.73(2.1)	26.9(2.3)	27.3(2.8)	27.0(2.3)
CHO	108.7(8.8)	26.54(1.8)	27.1(2.3)	27.6(2.3)	27.5(2.5)
Placebo	108.0(9.3)	26.8(2.0)	26.9(2.3)	27.10(2.3)	27.2(2.6)

Note: Data are expressed as mean (standard deviation) s: seconds; MTN: multi-ingredient; CHO: carbohydrate.

### Rate of perceived exertion (RPE)

**Fig 2:**
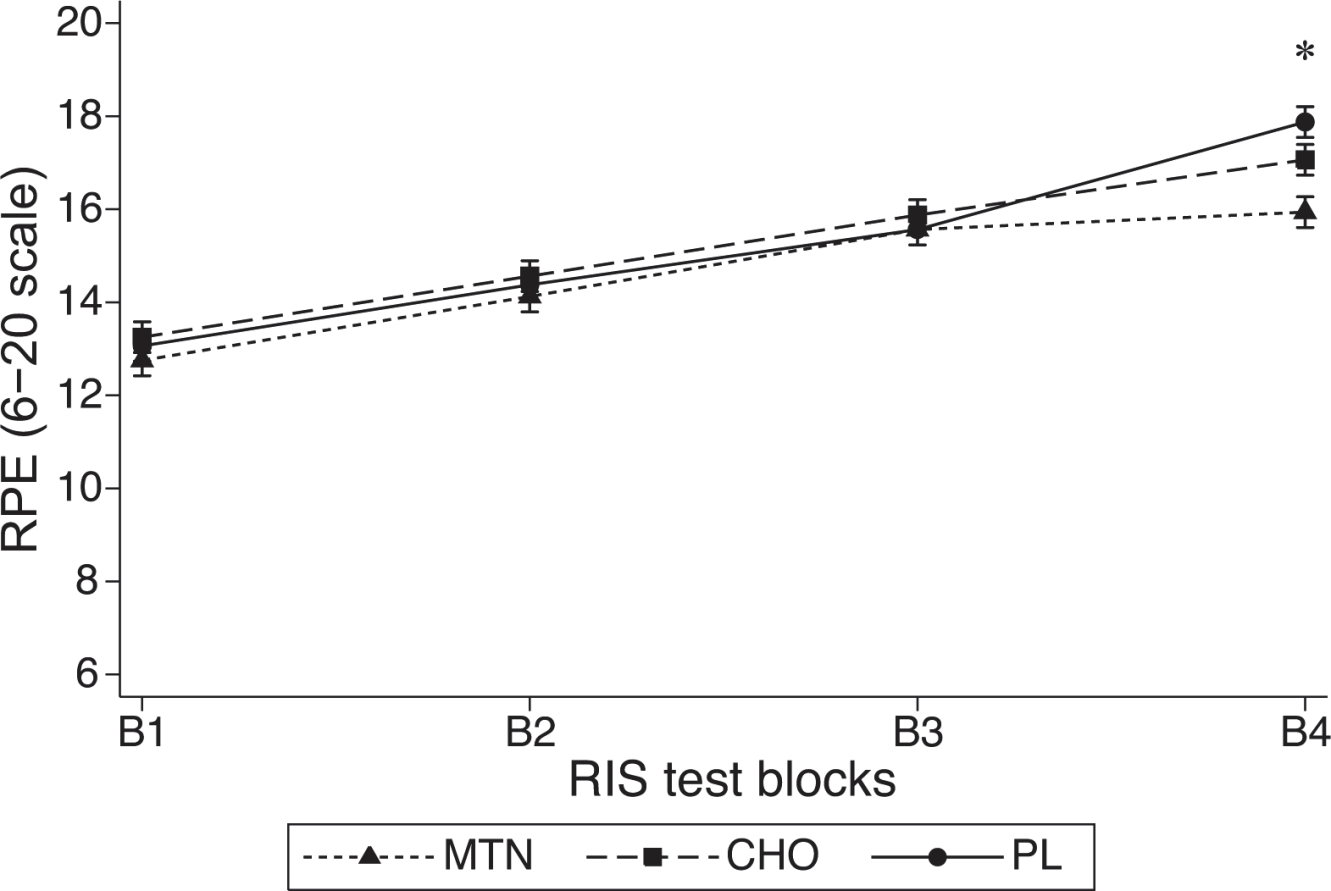
Rate of perceived exertion (RPE) values determined at the end of each block during the IRS test. *p>0.01 from MTN to both placebo and CHO at the end of the 4^th^ block.

A significant interaction effect was also observed between treatment conditions and blocks (F_6,90_ = 7.18, P<0.001, η^2^ = 0.02) in RPE. These values increased significantly throughout the exercise (Fig 2; F_3,45_ = 137.18, P<0.001, η^2^ = 0.41). Post hoc analysis revealed significant differences (P<0.001) between the first, second, third and fourth blocks for both the CHO (13.2±1.8; 14.6±1.9; 15.8±2.1; 17.1±1.9) and placebo (13.1±1.5; 14.4±1.8; 15.6±2.1; 17.8±1.4) conditions respectively. However under MTN, RPE showed different values between the first three blocks (P< = 0.001) but not between the 3^rd^ and 4^th^ block (12.7±1.7; 14.1±1.5; 15.6±1.6; 15.9±1.4). Even when no differences were observed between treatment conditions at blocks 1, 2 and 3, significantly (P<0.001) lower RPE values at block 4 were determined for MTN (15.9±1.4) compared with both placebo (17.8±1.4) and CHO (17.0±1.9) conditions.

### Fifteen-meter sprint test

Table 2 shows the 15-m sprints times measured at the 4 different time points (pre, post, 1h, and 24h) for the 3 analyzed conditions. A significant main effect was observed for time (pre, post, 1h, and 24h), (F_3,45_ = 29.92, P<0.001, η^2^ = 0.13), but not between treatment conditions (F_2,30_ = 0.67, P = 0.518, η^2^≈0). Post-hoc comparison between treatment conditions at each time point did not reveal significant differences.

**Table 2:** Fifteen-meter sprint times (s) measured at before (pre), immediately after (post), and 1 h (1h) and 24 h (24h) after intermittent repeated sprint test.

Condition	15 m (seconds)			
	pre	post	1h	24 h
MTN	2.44(0.20)	2.63(0.18)	2.61(0.20)	2.54(0.17)
CHO	2.43(0.17)	2.61(0.22)	2.66(0.23)	2.61(0.20)
Placebo	2.45(0.16)	2.58(0.19)	2.58(0.15)	2.57(0.18)

Note: Data are expressed as mean (standard deviation). MTN: multi-ingredient; CHO: carbohydrate.

Post hoc revealed significantly (P<0.01) longer sprint times for all treatment conditions at either post, 1h, and 24h compared with pre but not between the sprint times determined at the 3 post-time points (post, 1h, and 24h).

### Muscle damage markers

Table 3 shows the values determined for CK; Mb and IL-6 at the four different time points (pre, post, 1h, and 24h) for the treatment conditions. Before performing the IRS (pre), no significant differences treatment conditions were observed for the three analyzed markers. No significant main effects between treatment conditions (F_2,28_ = 1.41, P = 0.260, η^2^ = 0.02) was observed for CK. However significant effect of time points (pre, post, 1h, and 24h) was determined (F_3,42_ = 62.59 P<0.001, η^2^ = 0.40). Post hoc analysis revealed significant (P<0.05) higher values of CK at 24h compared with all other time points for all treatment conditions (Table 3).

**Table 3:** Muscle damage markers determined at before (pre), immediately after (post), and 1 h (1h) and 24 h (24h) after intermittent repeated sprint test.

Condition	CK (UL/L)				Mb (ng/ml)				IL-6 (pg/ml)			
	pre	post	1h	24 h	pre	post	1h	24 h	pre	post	1h	24 h
MTN	185.8±78.5	342.7 (95.2)	376.2 (114.6)	552.6 ^**ϑ**^ (285.0)	7.5(9.7)	302.0 (240.0)	265.4 (187.8)	4.7(7.8)	2.3(2.9	5.2(2.1)	5.5(3.5)	2.5(3.0)
CHO	169.9 (66.6)	279.8 (94.3)	302.4 128.4)	469.1 ^**ϑ**^ (206.8)	3.2(5.4)	203.5 (165.3)	241.8 (142.6)	8.9(15.2)	2.2(2.9)	4.5(2.1)	4.1(2.1)	2.0(2.5)
Placebo	144.1 (56.5)	296.4 (110.5)	360.7 (156.1)	589.9 ^**ϑ**^ (348.8)	14.4(18.6)	297.0 (292.6)	518.1* (255.2)	70.5*(80.5)	2.0(2.9)	5.0(2.4)	4.9(2.3)	1.8(2.4)

Note: Data are expressed as mean (standard deviation). MTN: multi-ingredient; CHO: carbohydrate; CK: creatine kinase; Mb, myoglobin; Interluekine-6: IL-6.

^**ϑ**^P<0.05 for CK levels measured at 24 h with respect to all other time points.

*P<0.05 from placebo to MTN and CHO.

Mb analysis showed significant interaction effects between treatment conditions and time (F_6,84_ = 4.83, P<0.001, η^2^ = 0.04), and differences between treatment conditions (F_2,28_ = 6.21, P = 0.005, η^2^ = .04), time (pre, post, 1h, and 24h), F_3,42_ = 43.04, P<0.001, η^2^ = 0.43).

**Fig 3:**
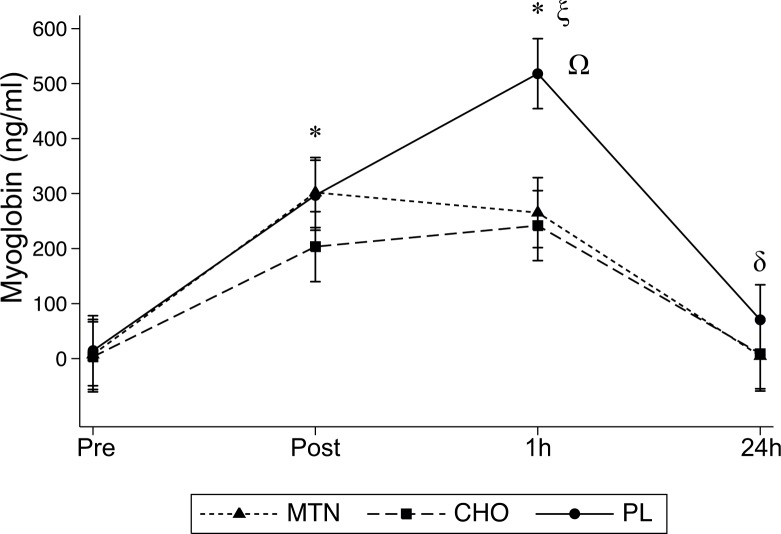
Serum Mb concentrations determined at all times points for the three conditions. *p<0.05 compared to pre for the three conditions; ξ p<0.01 from 1h to immediately after (post) only for placebo condition; δ p<0.01 compared to immediately after (post) for MTN and CHO; Ω p<0.05 at both 1hr and 24h post exercise from MTN and CHO to placebo.

Post hoc analysis revealed significantly higher Mb values for all 3 conditions at post (MTN and CHO P = 0.002; and placebo, P = 0.014) and 1h (P = 0.01 for the 3 conditions) compared with pre. However, only placebo showed significant higher values at 1h compared with post (P = 0.001). At 24h Mb returned to baseline levels in MTN and CHO conditions, reaching significantly lower values than those determined at post (MTN P = 0.002, CHO P = 0.003). In placebo, Mb significantly decreased from 1h to 24h (P<0.01) approaching baseline levels that still were not different from post. Additionally, significantly lower Mb values were determined at 1h and 24h for MTN (P = 0.016 and P = 0.025) and CHO (P = 0.011 and P = 0.015) compared with those determined after placebo condition respectively (Fig 3).

IL-6 analysis showed significant differences for time (pre, post, 1h, and 24h, (F_3,45_ = 42.73, P<0.001, η^2^ = 0.22) and condition effects (F_2,30_ = 3.96, p = 0.029, η^2^ = 0.009), but with no interaction effect with the time points (F_6,90_ = 1.93, p = 0.084, η^2^≈0). Post hoc analysis revealed higher IL-6 values for all 3 conditions at both post (MTN P = 0.001; CHO, P = 0.001; placebo, P = 0.003) and 1h (MTN, P<0.001; CHO, P = 0.001; placebo, P = 0.002) compared with pre. At 24h the IL-6 values approach baseline in all 3 conditions (MTN, CHO and placebo) being significantly (P = 0.01) lower than those measured at both immediately after and 1h following the IRS but not from those determined before the test.

### General Markers of immunity

Table 4 shows the values determined for Neutrophil, Lymphocytes and Monocytes at the 4 different time points (pre, post, 1h, and 24h) for the 3 conditions. Before performing the IRS, no significant differences between treatment conditions were observed for the 3 analyzed markers: Neutrophil (F_2,14_ = 1.08, P = 0.366), Lymphocyte (F_2,14_ = 2.44, P = 0.123) and Monocytes (F_2,14_ = 0.29, P = 0.754).

**Table 4:** General markers of immunity determined at before (pre), immediately after (post), and 1 h (1h) and 24 h (24h) after intermittent repeated sprint test.

Condition	Neutrophil (10^9^/L)				Monocyte (10^9^/L)				Lynphocytes (10^9^/L)			
	pre	post	1h	24 h	pre	post	1h	24 h	pre	post	1h	24 h
MTN	3.1(0.9)	3.8(1.4)	4.9(1.8)	3.1(1.1)	0.30(0.09)	0.36 (0.13)	0.42 (0.14)	0.33(0.09)	2.0(0.7)	2.0 (0.5)	1.8 (0.5)	1.8 (0.4)
CHO	2.9(1.1)	3.7(1.3)	4.0*(1.6)	2.9(0.9)	0.29(0.09)	0.34 (0.10)	0.36^§^ (0.09)	0.33(0.08)	1.8(0.5)	1.9 (0.4)	1.8 (0.4)	1.9 (0.3)
Placebo	2.8(0.7)	3.4(1.0)	4.5(1.6)	2.8(0.8)	0.31(0.08)	0.38 (0.11)	0.42 (0.12)	0.28(0.13)	1.9(0.7)	2.0 (0.7)	1.8 (0.4)	1.9 (0.5)

Note: Data are expressed as mean (standard deviation). MTN: multi-ingredient; CHO: carbohydrate *P<0.05 from CHO to MTN ^§^ P<0.05 from CHO to MTN and placebo.

**Fig 4:**
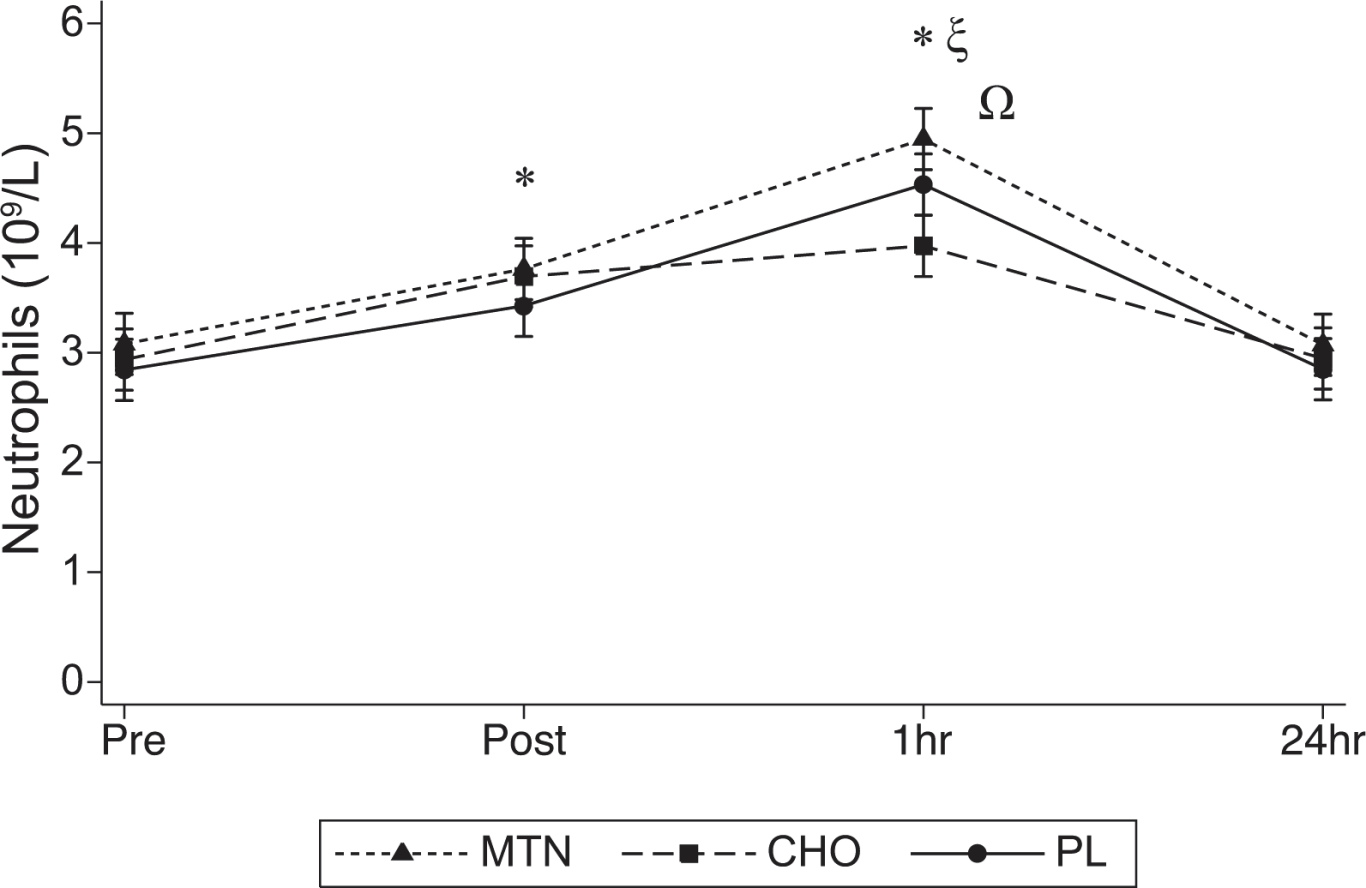
Neutrophil counts determined at all times points for the three tested conditions. *p<0.05 from post and 1h to pre for CHO and PL; ξ p<0.01 from 1h to pre for MTN and placebo; Ω p< 0.05 at 1h after exercise from MTN to CHO.

Neutrophil counts showed a significant interaction effect between treatment conditions and time (F_6,90_ = 2.77, P = 0.016, η^2^ = 0.01), but no significant main effect between treatment conditions (F = _2,30_ = 3.85, P = 0.051, η^2^ = 0.01). However, significant differences for time effects (pre, post, 1h, and 24h) were observed (F_3,45_ = 25.64, P<0.001, η^2^ = 0.21). Post hoc analysis revealed higher Neutrophil counts immediately after (post) compared to pre for CHO (P = 0.026) and placebo (P = 0.009) but not in the MTM condition. However, MTN and placebo showed higher Neutrophil concentration (P = 0.001) at 1h compared with pre. At 24h Neutrophils approached baseline values for all 3 conditions being significantly (P = 0.01) lower than those measured at 1h for MTN and placebo. Post hoc analysis also revealed significant lower Neutrophil concentration in CHO condition (P = 0.016) at 1h compared to MTN but not with placebo (Fig 4).

**Fig 5:**
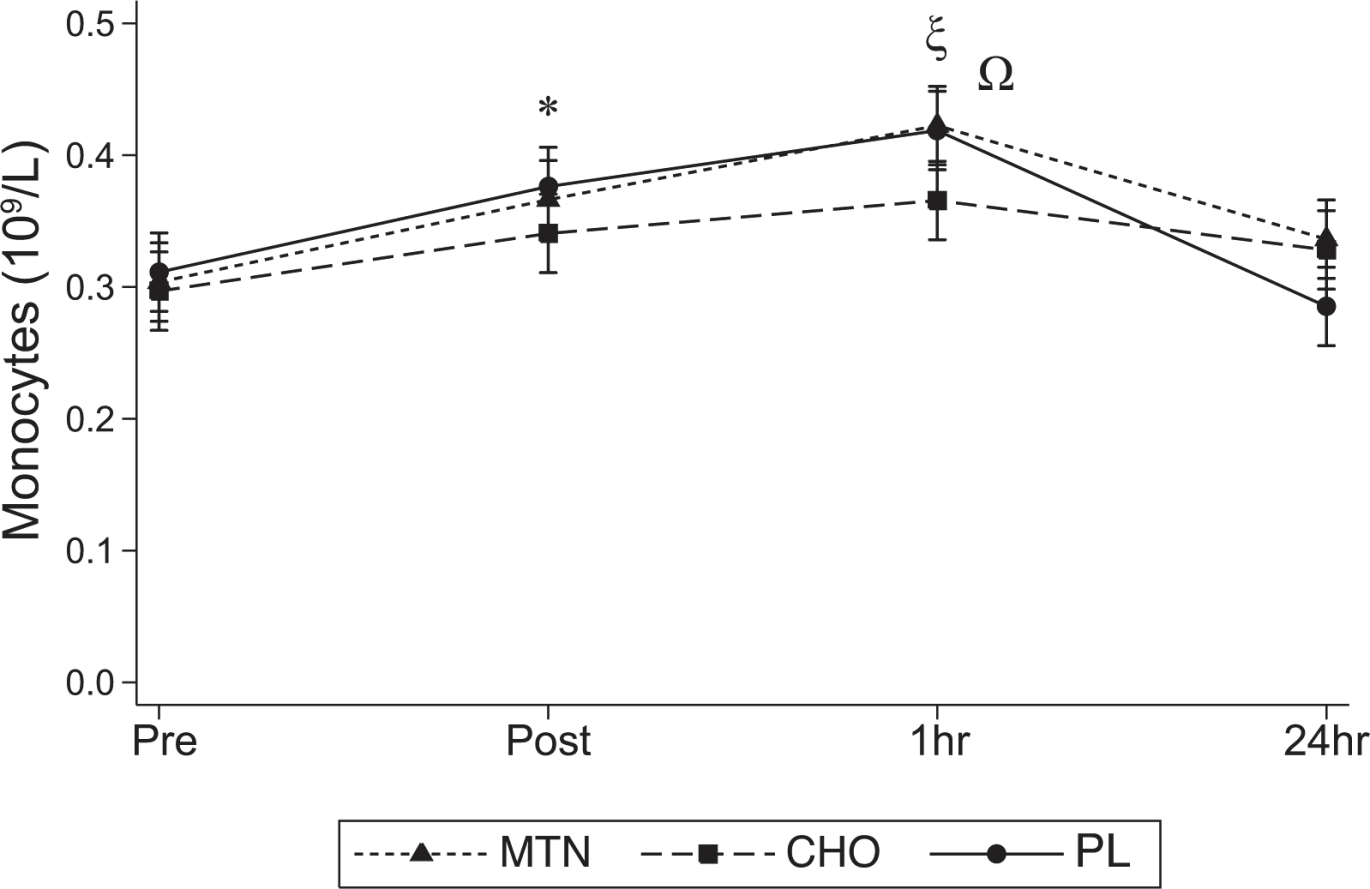
Monocytes counts determined at all times points for the three tested conditions. *p<0.05 from post to pre for MTN and PL ξ <0.05 from 1h to pre for all three conditions (MTN, CHO and placebo); Ω <0.05 at 1h after exercise from MTN and placebo to CHO.

Monocytes showed significant interaction effect between treatment conditions and time (F_6,90_ = 2.30, P = 0.041, η^2^ = 0.01). No significant main effect of treatment was found (F_2,30_ = 1.07, P = 0.356, η^2^ = .01). However, significant differences for time effects (pre, post, 1h, and 24h) were determined (F_3,45_ = 19.39, P<0.001, η^2^ = 0.12). Post hoc analysis revealed a higher Monocytes count at post compared with pre for MTN (P = 0.018) and placebo (P = 0.28) but not for the CHO condition. However, all 3 conditions showed higher Monocytes concentration at 1h compared with pre (MTN P = 0.002; CHO P = 0.003 and placebo P = 0.004). At 24h Monocytes decreased for MTN and placebo conditions being significantly lower than those measured at 1h (MTN P = 0.021; placebo P = 0.012). Post hoc analysis revealed a significant lower Monocyte concentration for CHO at 1h compared to both MTN (P = 0.020) and placebo (P = 0.018) (Fig 5).

No significant main or interaction effect was observed for Lymphocyte concentration.

## Discussion

The aim of our study was to examine functional (i.e., performance) indices related to a MTN ingestion schema. Supportive analyses were undertaken to detail immune and muscle damage indices related to muscular function. Overall, we found that the ingestion of a MTN or CHO supplement had no ergogenic or ergolytic affect on performance or patterns of serum creatine kinase and IL-6 responses over a 24h recovery period. Therefore, we reject our hypothesis that a MTN formula will improve performance under the parameters associated with our trial. We did, however, observe that ingesting MTN divided into four smaller intakes administered before and during a 90-min IRS plus another additional full dose ingested immediately after exercise was sufficient to attenuate the perception of fatigue experienced at the end of the 90-min IRS protocol. Although both MTN and CHO supplementation blunted the 1h-post exercise Mb increases, only CHO seems to be effective to attenuate the transitory 1h-post exercise neutrophilia and monocytosis commonly observed after heavy prolonged exercises [1]. Our results agree with those of Alghannam (2011) [7], who reported lower RPE values at the beginning and toward the end of a 90-min soccer-specific intermittent exercise when participants were supplemented with a similar carbohydrate-protein beverage prior and an interval synonymous with “halftime” compared to the ingestion of only carbohydrate or a low calorie placebo. In contrast to our findings, Alghannam reported a significant improvement during an exercise time-to-exhaustion test performed at 80% of V02max carried out just after a soccer-specific task, when the carbohydrate-protein mixture was administered.

Fatigue in intermittent sprint endurance activities can be caused by a number of factors, ranging from the generation of an inadequate motor command in the motor cortex (i.e. neural factors) to factors related to metabolite accumulation or energy supply [29]. The capacity to maintain a high level of performance while exercising at submaximal intensities closer to VO2max is limited by the amount of muscle glycogen available and the maintenance of higher levels of blood glucose toward the end of the task [7]. Conversely one single bout of a maximal 15-meters sprint performed after a 90-min IRS protocol would rely more on the ability to produce high levels of neural drive and the energy supply from the phosphagen system [29]. Consequently ingesting a nutritional supplement would be more effective to enhance performance at the post endurance run to fatigue exercise rather than on a single maximal 15-meter sprint run. In such as types of explosive short duration activities, regardless of the level of muscle glycogen or the amount of nutrient ingested during and after exercise, the progression of fatigue along long lasting intermittent activities leads to a failure to fully and maximally activate contracting musculature with a concomitant loss of force and sprint performance [30]. Indeed, other previous studies have observed similar drops in sprint performance, of about 2 to 9%, measured after intermittent sprints protocols [31]. In our study, we found that a multi-ingredient supplement had no effective attenuation for the reduction in sprint performance or the necessary speed up to reach the benchmark performance determined in the 15-meter sprint test.

The benefits of adding protein to carbohydrate on performance have shown to be evident when carbohydrate is provided at less than optimal rate of 60 g·h^-1^. However when exogenous carbohydrates are consumed at or above the optimal rate no further ergogenic effect has been reported by the ingestion of added proteins [22]. We administered two isoenergetic beverages providing a total of 53 g or 46 g of carbohydrate along a 90-min IRS protocol at CHO and MTN condition, respectively. Hence in both conditions the rate of carbohydrate delivered during exercise was beneath the optimal (46 g·h^-1^ and 35.5 g·h^-1^ respectively). Although of the addition of protein to a suboptimal amount of carbohydrate would be expected to improve performance, the energetic content of the supplements (~3.5 kcal·kg^−1^) may represent a limiting factor associated with the lack of any ergogenic effect observed during the IRS protocol [22].

Muscle glycogen depletion and dehydration observed during prolonged intermittent exercises would closely relate with a decrease in the exercise intensity [32]. Moreover, the perception of effort originated from the central nervous system would influence the intensity of exercise performed as fatigue progress along the workout or competition [33]. It has been postulated that the co-ingestion of carbohydrate-protein containing higher amount of branched-chain amino acids (BCAA) could reduce the ratings of perceived exertion, attenuate mental fatigue, and in some situations improve endurance performance [7] via a mechanism in accordance with the central fatigue hypothesis [34]. Results of our study would support the positive effect of added high quality protein to CHO to attenuate the rate of perceived exertion as the exercise approaches to the end (Fig 2), however no concomitant improvement in the intermittent endurance capacity was observed and therefore these results do not support the previously reported positive effects of carbohydrate-protein containing BCAA mixture to improve the intensity [8] enhance central drive and endurance capacity during prolonged exercises [7,35]. Perceived exertion has been proposed as useful to control pacing strategy in intermittent sport [33]. However, in our study athletes were allowed to self-select the intensity only during the 20 m walking whilst had to maximally sprint or perform at a relative pre-established sub-maximal intensity for the rest of the IRS test. Therefore, the proposed benefit of proteins added to carbohydrate for improving submaximal self selected exercise performance [8] would be expressed only at the central level with no impact on performance when exercising at pre established or maximal possible intensities. The exact mechanism by which the used MTN supplement has affected the perception of effort with no impact on the IRS performance warrants furthers investigations.

Regardless of the effect on performance, multi-ingredients supplementation has also been proposed as an effective countermeasure to attenuate exercise induced muscle damage [36] and the transitory post exercise immunosuppression [11]. Our results differ slightly from those reported by Naclerio et al [37] who observed positive effects of a multi-ingredient to blunt the rise in serum CK and Mb measured at 24h and 1 h respectively after performing a 90 min intermittent test in a sample of recreationally active team sport individuals. The present study showed inconsistent results, whereas CK peaks at 24h after exercise with no differences between treatment conditions, both MTN and CHO showed lower Mb values than placebo at 1h with no differences between them (Fig 3). Differences in the characteristic of the samples used in both studies (amateur soccer players vs recreationally practitioners from different team sports) or the high variability of the results would be the reason of such as the observed differences. In fact total CK is a highly variable, indirect and nonspecific biomarker of skeletal muscle disruption [38]. The analysis used for the assay of both CK and Mb does not distinguish between skeletal or cardiac muscle isoforms. In addition, substantial variation between participants (Table 3) together with the lack of homogeneity in the observed response for the three tested conditions makes difficult to identify any protective effects obtained from the ingestion of either MTN or CHO supplements. Furthermore, muscle damage, per se, is only a minor contributor to the rise of serum IL-6 during exercise. Indeed only sustained elevation in the IL-6 concentration observed from 6h to several days after exercise have been associated with the inflammatory response triggered by exercise induced muscle damage [39]. During prolonged exercise, active muscles secrete IL-6 in a hormone-like fashion to stimulate glucose output from the liver and lipolysis in adipose tissue when muscular glycogen becomes progressively depleted [40]. Thus, the patterns of IL-6 response observed in our study, increasing immediately after, 1h post and returned toward the baseline at 24h after exercise without differences between treatment conditions, would indicate that for our participants the 90-min IRS protocol was not hard enough to elicit both elevated levels of myofibrillar disruptions and to significantly deplete muscle glycogen to a level that would have been elicited higher IL-6 concentrations at least in the placebo condition. Moreover, exercise-evoked IL-6 may also act on the central nervous system to induce fatigue sensation [41] that has ultimately resulted in a significant decrement in endurance performance [42]. However, RPE values were lower only at the end of IRS during MTN condition. This result suggests that when glycogen is available, consuming a carbohydrate-protein based multi-ingredient supplement would attenuate the rise in fatigue perception even though no further effects on performance are achieved. Perhaps the 2h pre standardized meal containing 1 g·kg^−1^ CHO and 0.15 g·kg^−1^ protein, ingested by our participants was enough to prevent substantial glycogen depletion when this group of amateur soccer players performed a habitual 90-min intermittent activity.

On the other hand, CHO supplementation alone showed some effect to attenuate increases in blood neutrophil and monocyte counts observed at 1h post exercise (Figs 4 and 5). These results support the notion that only carbohydrate ingestion with no added proteins during and after heavy exercises would probably be the only effective scientifically supported nutritional countermeasure to exercise induced immune dysfunction [1]. Cells of the immune system have high metabolic rates and glucose availability can theoretically influence the immune response to exercise and the ability to maintain immunecompetence [43]. Additionally, a MTN combining CHO high quality proteins and enriched with L-glutamine, would have been expected to provide additional immunosuppression effects by counteracting exercise induced plasma glutamine depletion. However plasma glutamine concentrations following exercise do no decrease below threshold values that are detrimental to immune-function [11]. Thus, in ours previously well nourished athletes, the consumption of high quality proteins would not be effective to protect against exercise-induced immunosuppression. Furthermore the lower amount of carbohydrate provided in the MTN could have been the cause by which CHO and not MTN attenuated the increase in Neutrophil and monocyte counts.

The addition of L-glutamine and L-carnitine L-tartrate into the MTN formula does not appear to have any additional effect on the IRS performance or the 24h recovery period compared to CHO alone or placebo. There was also no effect on Mb levels since these were similar in both the MTN and CHO conditions. Even though the positive effects of L-glutamine supplementation on performance are not scientifically supported [44], some effects to prevent excessive muscle damage and neutrophil function suppression have been observed after several days of supplementation [45] but not after an acute intake as administered in the present study. In fact Neutrophil counts were higher in MTN compared to CHO at 1h post exercise (Fig 4). With regard to L-carnitine, its positive effects on exercise induced muscle damage and oxidative stress have been reported as a result of a three-weeks regular administration protocol [16,46] and not after an acute intake. Furthermore, as plasma L-carnitine peaks after 3 to 6h of ingestion [47], in order to promote its benefits, two doses where the last single intake should be administered around 3h before exercise has been recommended [47]. Thus, the protocol used in the present study where participants were supplemented immediately before, during and after exercise, would likely not favors some marketed positive effects of L-carnitine on performance, recovery and muscle damage.

A potential limitation of our study is that we only examined a 24h recovery period involving three time points and, therefore, is unable to generalize for longer periods of recovery time. In addition, we are unable to generalize to other sports that do not exhibit similar performance characteristics. Furthermore, other markers were not analyzed such as plasma aminoacidemia, glycaemia, serum glutamine and carnitine concentrations. We measured only general markers of immunity related to one aspect of innate immune function. Further studies should investigate the effect of feeding strategies on other aspects of immune function (e.g., lymphocyte proliferation and in vivo immune responses). Lastly blood IL-6 values do not allow estimating the autocrine and paracrine effects of this myokine within the skeletal muscle. To better analyze the IL-6 response to exercise, future investigations using microdialysis techniques or muscle biopsies should be considered. A strength of the present investigation is that we have implemented a randomized, counter balanced, cross over design using participants of similar training characteristics.

## Conclusions

The ingestion of a MTN (53g of CHO, 14.5g of whey protein, 1.5g of L-carnitine and 5g of L-glutamine) or CHO (69.5g maltodextrin) alone does not improve performance when ingested during and immediately after a 90-min intermittent repeated sprint test. Nor does this ingestion schema attenuate functional fatigue or alter CK and IL-6 responses measured immediately after, 1h, and 24h post exercise in amateur soccer players. Conversely, ingesting a MTN would be effective to attenuate fatigue perception compared to only CHO or placebo. Although both MTN and CHO appear to have some effects in blunting the Mb increases observed 1h post exercise, the high variability of our results makes difficult to consider a real positive effect of those supplements on muscle damage. Moreover only CHO with no added protein blunt the increase in Neutrophil and monocytes counts commonly observed after a 90-min intermitted repeated sprint protocol.

## Supporting Information

S1 Dataset Supporting information including all data collected during the study. (XLSX)
